# The Alar Fascia and Danger Space: A Modern Review

**DOI:** 10.7759/cureus.32871

**Published:** 2022-12-23

**Authors:** Tammy Phan, Johnson Lay, Frank Scali

**Affiliations:** 1 School of Medicine, California University of Science and Medicine, Colton, USA; 2 Anatomy and Medical Education, California University of Science and Medicine, Colton, USA

**Keywords:** mediastinitis, retropharyngeal space, prevertebral space, danger space, alar fascia

## Abstract

Purpose: Given the advancements in dissection modalities over the last decade, what is the current understanding of the alar fascia and its clinical implications as an access point into the danger space (DS)? The aim of the study is to provide an updated review of the alar fascia and danger space.

Methods: A comprehensive search of the alar fascia and danger space was performed through PubMed databases up to August 2022. Thirty-two sagittal E12 sheet plastination slices of the head and neck were analyzed under a stereomicroscope to assess the morphology and continuity of the retropharyngeal, alar, and prevertebral fasciae (PVF and their respective potential spaces).

Results: Recent advancements have provided evidence that the alar fascia is a true fascial layer between the retropharyngeal and danger spaces within the deep cervical region. Although its composition, histological features, and borders remain topics of controversy, the alar fascia is comprised of dense connective tissue and may serve as a physical barrier to prevent the spread of infection into the danger space. Complications arising from deep neck infections that invade the danger space include mediastinitis, necrotizing fasciitis, and empyema.

Conclusion: A proper understanding of the anatomy, structure, function, and potential spaces is crucial to assessing the alar fascia and danger space routinely in clinical practice, especially when imaging.

## Introduction and background

The alar fascia is classically described as one of the three deep cervical fascial (DCF) layers forming a boundary for the retropharyngeal and danger spaces (DSs) [1,2]. The existence of the alar fascia and the deep cervical spaces has been widely debated since their initial discovery. First described by Grodinsky and Holyoke in 1934, the alar fascia was characterized as a complete fascial layer intervening with the retropharyngeal and prevertebral fasciae (PVF) [3]. The term "alar" describes its "wing-like" appearance with borders that have been disputed by various authors investigating the deep cervical region [1-15]. Inconsistent descriptions of the alar fascia are likely due to challenges when attempting to dissect the retropharyngeal region using conventional measures, as true potential spaces may be obliterated in the process [9].

**Figure 1:**
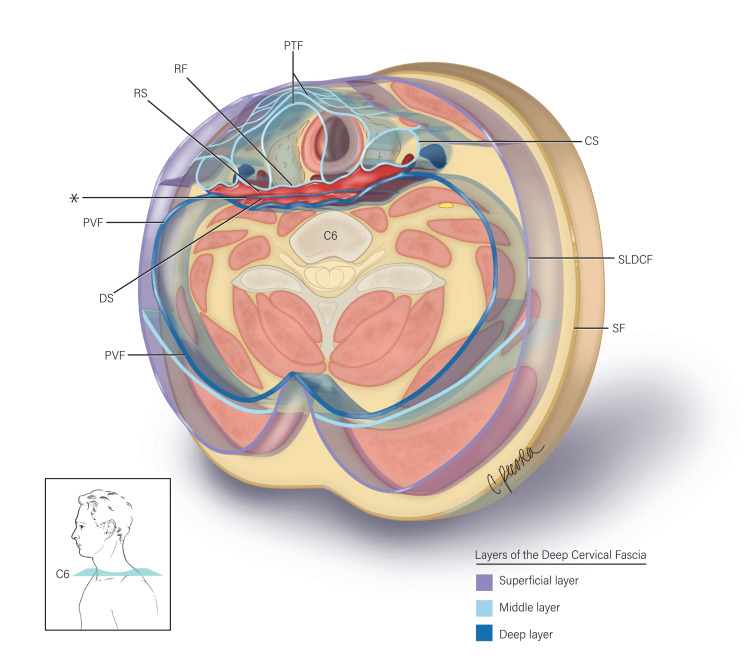
Illustration depicts a magnified transverse section at the C6 vertebral level. The three layers of the deep cervical fascia - superficial, middle, and deep (refer to legend) - are depicted in relation to the alar fascia (*). The alar fascia is situated between the retropharyngeal fascia (RF) anteriorly and the prevertebral fascia (PVF) posteriorly. Therefore, the alar fascia divides the potential space between the retropharyngeal and prevertebral layers to form the retropharyngeal space (RS) anteriorly and the danger space (DS) posteriorly. Also labeled: pretracheal fascia (PTF); carotid sheath (CS); superficial layer of the deep cervical fascia (SLDCF); superficial fascia (SF) (illustrated by Christina Pecora, MSMI).

The deep cervical fascia is classically defined by three layers: superficial, middle, and deep. The superficial layer of the deep cervical fascia (SLDCF) is a sheet of fibrous tissue that encircles the neck [3]. The middle layer consists of three distinct divisions: the sternohyoid-omohyoid, the sternothyroid-thyrohyoid, and the retropharyngeal layers [3]. The retropharyngeal layer is synonymous with the posterior visceral, retrovisceral, retroesophageal, buccopharyngeal, or visceral layer [3]. The deep layer consists of the alar fascia and the prevertebral fascia [1,3,9,14-16]. The alar fascia is also known as the intercarotid fascia, fascia alaris, and ala fascia [6]. The deep cervical fascial layers establish two clinically significant potential spaces: the retropharyngeal and danger spaces (Figure 1). These two spaces have been further explored, especially in the context of infection and its direction of spread.

Recent studies have found discrepancies in the borders, limits, histological composition, and function of the alar fascia. The alar fascia has been described as both loose fibroareolar tissue and dense connective tissue [1,6-8,12,13]. Traditionally, the alar fascia was described to begin at the base of the skull [2,3,15]. In contrast, Scali et al. identified the beginning of the alar fascia at the C1 vertebral level, which was later confirmed [7,11,12]. The inferior limit of the alar fascia has been identified at multiple vertebral levels, ranging from C6 to T2 [2,3,6,7,10-13].

The alar fascia is an important barrier that limits the spread of retropharyngeal abscesses and infections into the thorax through the danger space, leading to mediastinitis [12,13,17-19]. Surgically, the alar fascia is an important landmark when discussing retropharyngeal lymph node (RPLN) dissection and is an important fascial layer during preoperative planning of retropharyngeal lymphadenopathy [1,20]. The alar fascia also serves as a conduit for neurovasculature and functions to permit a sliding motion between the pharynx and esophagus during deglutition [11,13].

Proper diagnosis and treatment of certain head, neck, and mediastinal pathologies require a full understanding of the deep cervical fascia, including the alar fascia’s composition, contents, borders, and function. This study reviews the various nuances of the alar fascia in terms of its anatomy (gross dissection, sheet plastination, histology, and embryology), structure and function, potential spaces, and clinical implications. E12 plastinated specimens were carefully selected and analyzed to substantiate the most recent published findings. The advancements in understanding the alar fascia due to the advent of plastination and new anatomical findings have provided further insight into this "wing-like" structure, especially in its contribution to the danger space.

Methods

Literature Review

A relevant search of the PubMed database was performed to identify the relevant literature that pertained to the history and anatomy of the alar fascia and danger zone up to August 2022. The search terms included: "alar fascia," "danger space," "prevertebral space," "retropharyngeal space," "plastination," and/or "mediastinitis." The search was performed manually for eligible studies; all articles were requested and obtained via the California University of Science and Medicine (CUSM) Information Commons (Library) Interlibrary loan system. All appropriate literature was organized and analyzed chronologically to represent a current understanding of the alar fascia. All included studies described the alar fascia and danger space as seen in cadaveric specimens, imaging (computed tomography and magnetic resonance imaging), and relevant clinical case reports.

Data Extraction

Data were extracted from selected studies set forth by the authors to represent the most up-to-date understanding of the alar fascia and danger space in the literature. The data included details on these two topics concerning their anatomical and clinical relevance. The data extracted encompassed embryology, histology, gross dissection, and sheet plastination. The retrieved data were evaluated and selected based on the study methods, a timeline with the introduction of technological and study advancements, such as plastination techniques and imaging, and clinical reliability.

Sheet Plastination

Plastination was prepared and obtained from prime prosections. Sixteen 4 mm-thick translucent sheet plastinates were examined on a radiographic light-emitting diode lightbox. Each slice was subsequently reviewed macroscopically under low magnification (range 0.63× to 1.25×) with a Leica MZ8 Stereoscopic Dissecting Microscope (Leica Microsystems, Inc., Buffalo Grove, IL, USA). The alar fascia and its potential spaces were examined, measured, and photographed. The superior attachment site of the alar fascia was examined along the sagittal and parasagittal planes. Specimens with signs of variation, prior surgery, or trauma in the region of interest were excluded from this study. Measurements were recorded using a Neiko 01407A Electronic Digital Caliper (range 0-150 mm; resolution 0.01 mm; accuracy ±0.02 mm; Neiko Tools, China). Photographs were captured with a Canon EOS 6D Digital Camera (Canon, Inc., Ōta, Tokyo, Japan). All guidelines for the use of cadaveric material were followed.

## Review

Anatomy

Deep Cervical Fascia and Its Relation to the Alar Fascia

The DCF has traditionally been presented as three layers: superficial, middle, and deep [3,4,16,21]. The superficial layer of the DCF is composed of a continuous fibrous sheet encircling the neck, which begins at the base of the skull and extends to the thoracic inlet [4,9,14]. The DCF’s middle layer is composed of the retropharyngeal fascia and encompasses the thyroid gland, trachea, and esophagus [21]. The retropharyngeal fascia’s borders are composed of the thyroid cartilage and hyoid bone anteriorly and the base of the skull posteriorly [21]. The retropharyngeal fascia fuses with the alar fascia posteriorly and is continuous with the fibrous pericardium inferiorly [21].

Alar Fascia - Anatomical Boundaries and Limits

The alar fascia is commonly described as a complete fascial layer that lies coronally between the retropharyngeal and prevertebral fasciae and extends between the medial aspect of the carotid sheaths to form the anteromedial sleeve of the carotid sheath [1-3,6,7,12,13,22]. Posteriorly, the alar fascia coalesces with the prevertebral fascia at the tips of the cervical transverse processes and then courses posteriorly to form the posterior and lateral walls of the carotid sheath [3,13,21]. Multiple studies have described the alar fascia adhering to the prevertebral fascia at the midsagittal line [7,23]. Variations have been noted in which the alar fascia fuses with the retropharyngeal fascia as it crosses the median plane between the carotid sheaths bilaterally [6,7].

Although the alar fascia’s existence has been verified, its anatomical borders and limits have been inconsistently presented. Initially, the alar fascia was identified at the base of the skull [2,3]. Other studies have demonstrated the beginnings of the alar fascia at the C1 vertebrae [1,7,8,11,12]. Using E12 sheet plastination, Scali et al. identified the beginnings of the alar fascia at the C1 vertebral level in situ [12]. Between the inferior nuchal line and the base of the skull, the authors noted disordered soft tissue tracts and loose fibroareolar tissue [12]. The inferior limit of the alar fascia has been identified between C6 vertebrae and T2 vertebrae through different dissection methods [2,3,6,7,10,11,13]. The alar fascia was found caudally at the C6 and C7 vertebrae [6]. Grodinsky and Holyoke [2] and Standring [3] identified the inferior boundary at the C7 vertebrae, where it fused with the retropharyngeal fascia. Various studies identified the alar fascia as being continuous with the retropharyngeal fascia at T2 vertebrae, while Levitt vaguely pinpointed T1-T2 vertebrae [7,10,13]. In comparison, embryological studies identified the inferior limit at the T1 vertebrae [11].

Alar Fascia - Further Insight Through Plastination and Other Techniques

Compared to traditional cadaveric dissections, plastination offers valuable insight into detailing the in-situ arrangement of fascial layers and potential spaces [12,24,25]. During the process of plastination, soft tissue structures shrink, allowing for easy visualization of the fascial plane that is otherwise lost by conventional dissection measures [24]. Sheet plastinates can be observed under magnification to identify the structure of interest and its correlating histology [26]. Prior to the work of Scali et al., the alar fascia was never described in this manner; these findings were later verified by multiple follow-up studies [6,8,12,13,27]. Utilizing conventional dissection, the alar fascia was identified as a distinct layer of the DCF, starting at the C1 vertebrae, posterior to the retropharyngeal fascia [7,8]. Above the C1 vertebrae, the alar fascia was found joining the fascia surrounding the longus capitis and, therefore, did not attach to the skull base [8]. Correspondingly, in an embryological study by López-Fernández et al., the alar fascia was identified at the C1 vertebrae in human specimens at 6-12 weeks of gestation [11]. In contrast, a recent histological study described the alar fascia as an extension to the basilar portion of the occiput with a loose attachment in the midline to the anterior arch of the atlas bone [13]. Similarly, in cadaveric dissections, the alar fascia was identified as extending cranially to the base of the skull [6].

Substantiation of Current Findings With Plastinated Cadaveric Specimens

**Figure 2:**
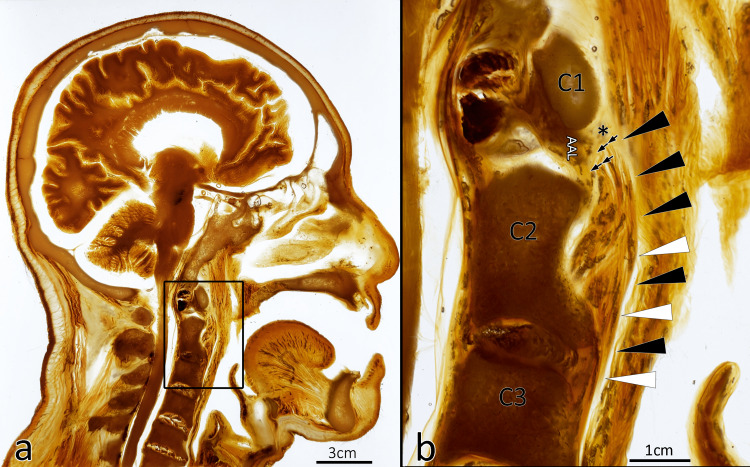
Single 4 mm translucent sheet plastinate of the head and neck revealing the relationship of the alar fascia with its respective surrounding structures and potential spaces. (a) E12 plastinated sagittal section of the head and neck (4-mm-thick section) with call out revealing the area of interest (scale bar = 3 cm). (b) Magnified region of anatomy between the first (C1) and third (C3) cervical vertebral levels. A dense membrane, identified as the alar fascia (black arrowheads), extends from the inferior pole (*) of the anterior arch of the atlas. The alar fascia separates the retropharyngeal space from the danger space. The anteroinferior attachment point diverges posteriorly (double arrows) to coalesce with the anterior ligaments of C2 and the atlantoaxial ligaments (AAL) (scale bar = 1 cm).

Examination of the 4 mm sagittal sheet plastinates revealed that the alar fascia converges and fuses with the prevertebral fascia superiorly and attaches as a single dense ligamentous band to the anterior tubercle of the anteroinferior arch of the atlas. These ligamentous bands diverge posteroinferiorly and posterosuperiorly from their atlas attachment to coalesce and blend with the anterior longitudinal ligament and the atlantoaxial ligaments (ALLs) (Figure 2). The superolateral fibers of the alar fascia create a haphazard arrangement as it breaches the region between the C2 vertebral body and the anterior arch of C1. Along the parasagittal plane, the terminal attachment points could not be identified, but the alar fascia fuses with the atlantoaxial ligaments.

Sheet plastination in this study was mainly used to confirm the description of the retropharyngeal fasciae/space morphology but has revealed an unreported finding regarding the alar fascia’s superior attachment site. Superiorly, the alar fascia converges and fuses with the prevertebral fascia as a single dense ligamentous band of tissue to the anteroinferior arch of the atlas. As what appeared to be a single band of tissue, this ligamentous structure diverges posteroinferiorly and posterosuperiorly to blend with the anterior longitudinal ligament and the atlantoaxial ligaments. The results of this plastination finding require further investigation with a larger sample size. Histology and confocal microscopy would be useful modalities to detail the true superior attachments of the alar fascia and its relationship with nearby anatomical structures.

Embryological development

The embryological origin of the alar fascia provides insight regarding its final composition, borders, and potential functions. At week six of gestation, the alar fascia primordium was identified in the retropharyngeal space at C7 to T1 vertebral levels [11]. The alar fascia primordium was notably attached to the retropharyngeal primordium and was composed of mesenchymal cells and collagen fibers [11]. During the seventh and eighth weeks of gestation, the alar fascia primordium migrates superiorly to the C2-C3 vertebral level at the midline and extends laterally to the superior cervical ganglion [11]. By week nine, the retropharyngeal, alar, and prevertebral fasciae can be identified as distinct layers, with the retropharyngeal and prevertebral fasciae being thicker than the alar fascia [11]. These findings reinforced earlier studies that identified the alar fascia during weeks nine to twelve of gestation [28]. By week 15, the alar fascia terminated laterally at the adventitia, which would develop into the carotid sheath [29]. During weeks 18 to 25 of gestation, the alar fascia extends laterally and connects to the cupula pleurae and upper portions of the parietal pleura, contributing to the primitive form of Sibson’s fascia [29]. Thus, the alar fascia’s development is thought to initially be a band connecting the bilateral common carotid arteries, later obtaining attachments to the mediastinal great vessels, trachea, esophagus, and suprapleural membrane [29].

Structure and function of the alar fascia

The alar fascia’s composition and thickness have been a subject of controversy [1,8]. The alar fascia’s composition has commonly been described as loose fibroareolar tissue compared to the retropharyngeal and prevertebral fasciae [1,7,8,13]. Conversely, Scali et al. utilized manual traction to demonstrate the alar fascia’s resistance, thickness, and integrity to be comparable to that of the retropharyngeal and prevertebral fasciae [12]. In concordance, a cadaveric dissection demonstrated the alar fascia as a partially thick wall that is composed of dense regular connective tissue [6]. Histological findings revealed collagen fibers in the retropharyngeal, alar, and prevertebral fascial layers [7]. Therefore, it is reasonable to suspect the alar fascia’s structural integrity may function as a physical barrier preventing the spread of retropharyngeal infections into the danger space [7,12,13].

There are various theories in terms of the alar fascia’s function. The alar fascia may permit the sliding of the pharynx and esophagus during deglutition [11]. This is supported by the low friction interface and lack of neurovasculature between the retropharyngeal and alar fasciae [6,13]. The alar fascia also isolates the cervical viscera from the vertebral column to permit the expansion of the esophagus during deglutition and coughing [13]. Moreover, the alar fascia may serve as a stable conduit for neurovasculature [1,13,30]. The alar fascia is one of the deep cervical fascial layers that contribute to the carotid sheath [1,4,9,10,14,16]. In a cadaveric study, the recurrent laryngeal nerve was found to pierce through the alar fascia from the carotid sheath’s inner edge [1,30]. The sympathetic trunk was also identified within the fibroareolar tissue of the alar fascia [1]. S100 and alpha-SMA protein staining demonstrated that the alar fascia is well vascularized and innervated [13]. A Masson trichrome stain of the alar fascia illustrates its contributions to the carotid sheath’s medial and anterior walls [7]. Since the alar fascia has a significant contribution to the carotid sheath’s morphology, it may serve to support and retain the carotid sheath and its contents in place [13].

Potential spaces

Retropharyngeal and Danger Spaces - Orientation

The alar fascia occupies the space between the retropharyngeal and prevertebral fasciae creating two potential spaces: the retropharyngeal and danger spaces [1-3,7,9,12,22]. The two distinct potential spaces are defined by their anatomical limits, content, and associated diseases. The borders of the retropharyngeal space have been inconsistently presented [1]. The retropharyngeal space was historically described as a potential areolar space between the retropharyngeal fascia anteriorly, carotid sheath laterally, and alar fascia posteriorly [3,16,20,31]. Other studies have disputed that the retropharyngeal space’s lateral border is composed of the alar fascia [14,32]. Granich et al. describe the retropharyngeal fascia fusing with the alar fascia to form the lateral border [33]. However, in a histological study of the deep neck fascia, there was no sagittal connection between the retropharyngeal and prevertebral fasciae [7]. In studies where the alar fascia is not identified as a fascial layer, the retropharyngeal space is considered a potential space containing loose areolar tissue and is bound by the retropharyngeal fascia anteriorly, the prevertebral fascia posteriorly, and the carotid sheath posterolaterally [4]. The retropharyngeal space extends from the base of the skull to conflicting inferior limits: vertebral levels ranging from C6 to T4, C6 to T6, T1, T2, T4, and the mediastinum [3-5,16,20,22,23,32-36].

Retropharyngeal and Danger Spaces - Characterization

A midline raphe divides the retropharyngeal space into two halves [35]. The retropharyngeal fascia is fused with the alar fascia at the midline [16]. In a study performed by Grodinsky and Holyoke, adult cadavers were injected with gelatin colored by Indian ink; the gelatin dye rarely ruptured into the pretracheal and danger spaces due to the tight adhesions between the retropharyngeal and alar fasciae [3]. The retropharyngeal space’s contents include adipose tissue and the retropharyngeal lymph nodes [1,5,14,20,22,23,35,37,38]. On each side of the retropharyngeal space, there are two chains of retropharyngeal lymph nodes: medial and lateral [14,22,35,38]. The lateral retropharyngeal lymph nodes can be identified as medial relative to the internal carotid artery at the C1-C2 vertebral levels [37].

The danger space is posterior to the retropharyngeal space. It has also been referred to as the prevertebral, anterior visceral, or vascular visceral space [39]. The danger space is comprised of loose areolar tissue defined by the alar fascia anteriorly and the prevertebral fascia posteriorly [2,9,16]. The tips of the transverse processes make up the lateral limits [3,9,10,33]. The danger space is sealed off through the fusion of the retropharyngeal and prevertebral fasciae with the endothoracic fascia [13]. The danger space extends from the base of the skull through the posterior mediastinum to the diaphragm [2,3,9,10,14-16,22,23,32,34,36]. Utilizing latex injection, the danger space was visible posterior to the alar fascia and extended inferiorly to the posterior mediastinum [6]. On the other hand, the danger space contains only adipose tissue [23,40].

Clinical implications

Oropharyngeal Infections and the Role of the Alar Fascia

**Figure 3:**
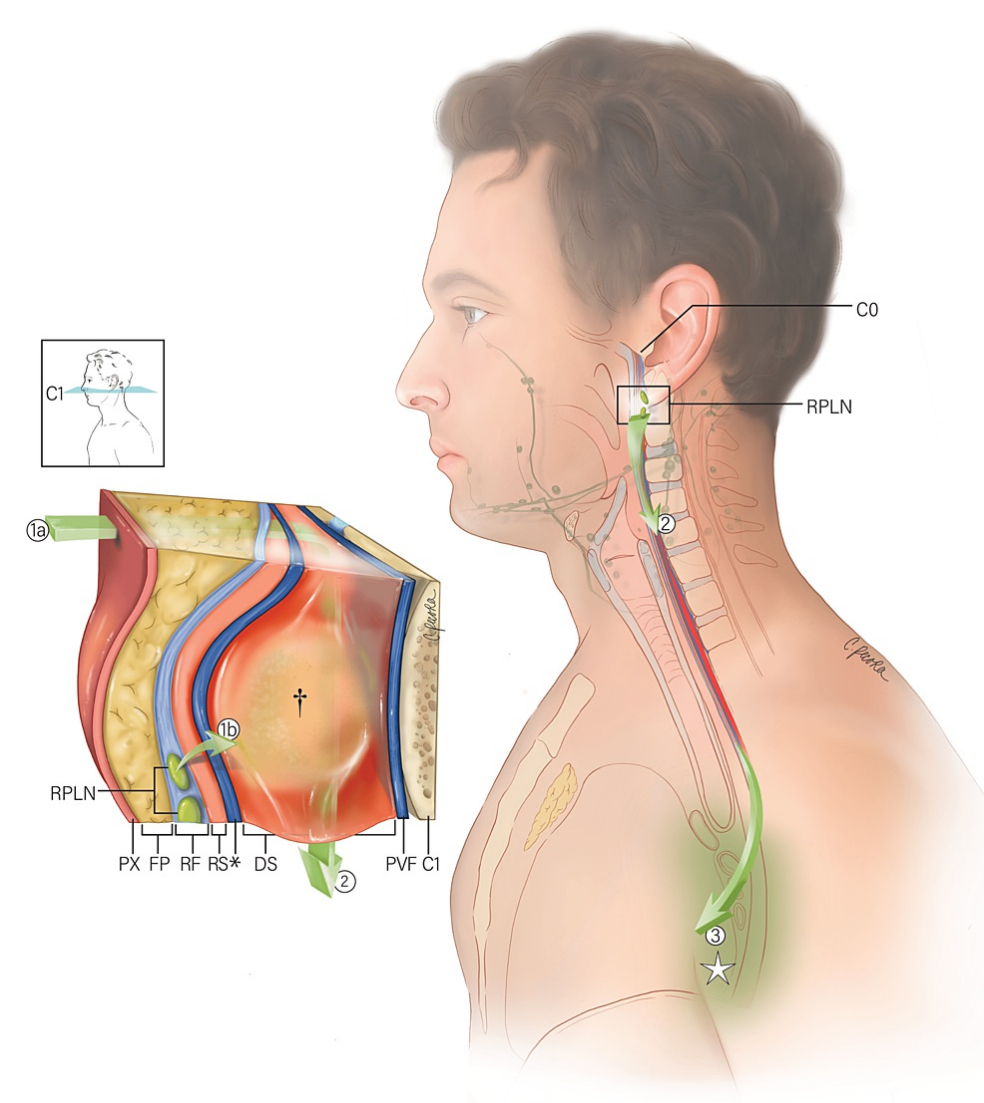
Illustration depicting the route of a retropharyngeal abscess and an oropharyngeal infection. An en bloc section of the soft tissue structures is depicted at the C1 vertebral level (see call-out). Pharyngeal infection may breach the retropharyngeal fascia (RF), retropharyngeal space (RS), and alar fascia (*), entering the danger space (DS) (1a). Alternatively, an oropharyngeal infection may drain into the retropharyngeal lymph nodes (RPLN), located on the lateral sides of the retropharyngeal space; the infection may then breach the alar fascia resulting in an abscess formation (†) in the danger space (1b). From the danger space, the infection can pass inferiorly (2) to the posterior mediastinum (☆) (3). Also labeled: posterior wall of the pharynx (PX); retropharyngeal fat pad (FP); prevertebral fascia (PVF); skull base (C0) (Illustrated by Christina Pecora, MSMI).

The different fascial planes of the neck play an essential role in limiting the spread of disease [9,10,16,41]. The anatomy of the DCF is important when attempting to understand the pathology and spread of disease into the mediastinum [4,9,16,22,23,32,33,36]. The alar fascia is a key landmark in surgical procedures [1,13,30]. Oropharyngeal infections drain into the retropharyngeal lymph nodes and may form an abscess [9,10,31,42]. Pathology in the retropharyngeal space may present symmetrically or asymmetrically due to the midline raphe that separates the retropharyngeal space into two halves [23,35]. Retropharyngeal abscesses may cause complications such as spontaneous rupture into the airway with aspiration, laryngeal spasm, bronchial erosion, septicemia, metastatic abscesses, thrombosis of the jugular vein, hemorrhage, or mediastinitis [10]. As a result, dissection of retropharyngeal lymph nodes may be required for proper management. The alar fascia is also an important anatomical guide when identifying the retropharyngeal lymph nodes [1]. The retropharyngeal lymph nodes can be located medial to the internal carotid artery and sympathetic trunk and are anterior to the alar fascia at the level of the transverse process of the atlas [1]. Moreover, the alar fascia can be verified by the sympathetic trunk, which passes within its fibroareolar substance [1]. In a transoral excision, the retropharyngeal lymph nodes can be exposed within the retropharyngeal space on the alar fascia [20]. The alar fascia is also an important structure to note to avoid damage to the recurrent laryngeal nerve during anterior cervical spine surgery [30]. Therefore, it is important to identify and preserve the alar fascia during surgical procedures (Figure 3).

Deep Neck Infections in the Carotid Sheath

The carotid sheath is an important anatomical landmark utilized to drain deep neck infections [1,10]. Infectious processes in the carotid sheath rarely occur since there is minimal areolar connective tissue between the vessels [4,9,10,16]. When pathology occurs, it tends to be localized [4,9]. However, the DCF is continuous with the carotid sheath, which encircles neurovascular structures within the carotid space; localized infections within the DCF may therefore spread into the carotid canal ("Lincoln’s Highway"), affecting its contents and structures along its pathway [12,16,43].

Infections Into the Danger Space and Its Complications

The danger space has various clinical implications. Infections within the danger space usually occur as extensions from other potential spaces [9,16]. A retropharyngeal abscess can descend into the superior mediastinum or penetrate the alar fascia and enter the danger space [9,21,32]. The alar fascia is an important boundary in the establishment of the danger space. Breach of the alar fascia by infectious agents or metastasis may lead to the spread of disease into the posterior mediastinum, resulting in an increased risk for morbidity and mortality [44]. Various complications can result, including hemorrhage, spontaneous rupture into the airway with aspiration, laryngeal spasm, bronchial erosion, septicemia, metastatic abscesses, thrombosis of the jugular vein, and mediastinitis [10].

Mediastinitis

Deep neck space abscesses can cause significant morbidity in children, as the complication rate can be as high as 9.4% [45]. On the other hand, deep neck infections in adults may involve multiple potential spaces and lead to more lethal consequences [44]. Head and neck infections may drain into the parapharyngeal space, spread to the retropharyngeal space, invade the danger space (which serves as a conduit to the inferior mediastinum and pericardium), and lead to mediastinitis [34]. The parapharyngeal space, also known as the lateral pharyngeal space, is an inverted-pyramid region that extends from the base of the skull to the hyoid bone and is intimately related to the retropharyngeal space posteriorly [14,16]. The spread of infection into the retropharyngeal, danger, and prevertebral spaces is considered life-threatening because it can spread directly into the superior mediastinum or invade the danger space and enter the posterior mediastinum [36]. Although mediastinitis is a rare complication, the mortality rate, which was as high as 50% in the pre-antibiotic era, remains high at 40% in the post-antibiotic era [27,46].

Retropharyngeal abscesses can spread into the danger space by breaching the alar fascia [18,19,21,32]. Chong et al. presented a case of adult epiglottitis that led to mediastinitis and bilateral thoracic empyema [43]. It was proposed that the infection began in the supraglottic area, spread to the parapharyngeal space, entered the retropharyngeal space, ruptured into the danger space, and traveled inferiorly to the diaphragm [43]. At the level of the diaphragm, the infection can spread into the posterior mediastinum, causing empyema and rupturing the parietal pleura into the pleural space [43].

Descending necrotizing mediastinitis is a lethal infection that must be diagnosed and treated early [18]. The mortality rate of descending necrotizing mediastinitis has been reported to be 25-40% [47]. A deep neck space infection can involve the parapharyngeal space followed by the retropharyngeal space, resulting in complications such as mediastinitis, cervical necrotizing fasciitis, and intracranial extension [48]. Conversely, a pneumomediastinum case may begin as an alveolar rupture and progress through the pulmonary vasculature, mediastinum, danger space, retropharyngeal space, and parapharyngeal space [33]. Furthermore, delayed diagnosis and inadequate mediastinal drainage in deep necrotizing mediastinitis have a higher risk for mortality; a more aggressive transthoracic approach through a standard thoracotomy procedure may be warranted, regardless of mediastinal involvement [49]. Therefore, it is important to understand the fascial relationships, potential spaces, and possible routes of infection to discern the pathological process as well as potential and proper surgical management [18,23,44,49].

Diagnostic Imaging and the Search for the Alar Fascia

Computed tomography (CT) and magnetic resonance imaging (MRI) are recognized as the primary modalities for the diagnosis and proper management of retropharyngeal infections [31,41]. Although they are separate potential spaces, the retropharyngeal and danger spaces are erroneously considered as one because their separation, formed by the alar fascia, cannot be visualized on imaging [14,23,40]. In two case studies, the retropharyngeal space was identified between pharyngeal constrictor muscles and the prevertebral muscles posteriorly on imaging [35]. Therefore, it was suggested that the retropharyngeal and danger spaces be considered a single potential space [40]. In contrast, Mukherji and Castillo identified the alar fascia in a case of edema within the retropharyngeal space on axial CT imaging [38]. Additionally, in an imaging study of 137 subjects with normal CTs, 72% of the persons’ alar fascia adipose tissue was identifiable [50]. The presence of the alar fascia distinguishes the retropharyngeal space and danger space as two separate potential spaces. The discrepancies in identifying anatomical structures on imaging demonstrate its lack of reliability for diagnosing deep neck infections [32]. With these advancements in technology, it is important to recognize the potential spaces within the head and neck to accurately detect, diagnose, and understand the scale and scope of disease.

Reimagining the alar fascia in a modern light

Grodinsky and Holyoke extensively described the fasciae and fascial spaces of the head and neck region [3]. Follow-up studies demonstrated the alar fascia as a distinct layer through various methods, including cadaveric dissections, histological studies, embryological studies, and sheet plastination [6,7,11,13,28-30]. From these recent developments, the alar fascia is reimagined in a modern light that provides further insight into its clinical relevance in the danger space.

Based on the current literature, future studies should focus on isolating and defining the true integrity and morphology of the alar fascia. Confocal microscopy can be a useful measure to delineate the layers and spaces of the retropharyngeal region in situ. This technique can offer further insight into the relationship between these various layers by detecting autofluorescence from collagen fibers [24]. Specifically, it can be performed without compromising the integrity of the separate layers. In addition, multiple studies have determined that the alar fascia is composed of collagen but does not provide a specific type [7,11]. Defining the alar fascia’s collagen subtype would determine its integrity and thus clarify its function and the organization of the retropharyngeal spaces and related structures.

Given these new findings on sagittal E12 sheet plastination, further investigation of the alar fascia’s superior attachment is warranted to understand the fascial relationships and potential access points for infection to breach into the danger space. The alar fascia is a key landmark in surgical procedures, as it constitutes the anterior border of the danger space [1,13,30].

## Conclusions

From an anatomical standpoint, the alar fascia can be visualized through the lenses of gross dissection, sheet plastination, histology, and embryology. These techniques offer insight into the complexity of understanding what the alar fascia is and what role it plays in the human body. Further exploration of the alar fascia is warranted for a better understanding of its true morphology and clinical implications in the spread of infection into the danger space. Specifically, such studies can investigate the superior attachment of the alar fascia utilizing multiple modalities. The alar fascia requires special consideration in surgical procedures due to its location in the head and neck. Since it originates in the C1-C2 region, the alar fascia may be implicated in various diseases, such as Down syndrome and rheumatological diseases.

When discussing the alar fascia, the question is no longer whether it exists but rather how it can be identified radiographically. From a clinical standpoint, new technologies and modalities, such as contrast-enhanced CT and MR imaging, have allowed for a more accurate diagnosis of the alar fascia’s associated pathologies, especially infections involving the danger space. Clinicians, including radiologists, should include the alar fascia and its related spaces in their diagnostic workup. By identifying the alar fascia, clinicians are able to recognize its value in surgical and diagnostic applications. Most importantly, patient outcomes may improve with consideration given to head and neck surgery, deep neck infections, retropharyngeal abscesses, and mediastinitis.
